# RNAi-Mediated Gene Silencing of *Trcot1* Induces a Hyperbranching Phenotype in *Trichoderma reesei*

**DOI:** 10.4014/jmb.1909.09050

**Published:** 2019-11-18

**Authors:** Fei Gao Mengzhu Li, Weiquan Liu, Yingguo Bai, Tu Tao, Yuan Wang, Jie Zhang, Huiying Luo, Bin Yao, Huoqing Huang, Xiaoyun Su

**Affiliations:** 1 Key Laboratory for Feed Biotechnology of the Ministry of Agriculture, Feed Research Institute, Chinese Academy of Agricultural Sciences, Beijing 0008, P.R. China; 2 College of Biological Sciences, China Agricultural University, Beijing 100193, P.R, China

**Keywords:** *Trichoderma reesei*, *Trcot1*, hyperbranching, RNAi, cellulase, biofuel

## Abstract

*Trichoderma reesei* is the major filamentous fungus used to produce cellulase and there is huge interest in promoting its ability to produce higher titers of cellulase. Among the many factors affecting cellulase production in *T. reesei*, the mycelial phenotype is important but seldom studied. Herein, a close homolog of the *Neurospora crassa* COT1 kinase was discovered in *T. reesei* and designated *Tr*COT1, which is of 83.3% amino acid sequence identity. Functional disruption of *Trcot1* in *T. reesei* by RNAi-mediated gene silencing resulted in retarded sporulation on potato dextrose agar and dwarfed colonies on minimal medium agar plates containing glucose, xylan, lactose, xylose, or glycerol as the sole carbon source. The representative mutant strain, SUS2/Trcot1i, also displayed reduced mycelia accumulation but hyperbranching in the MM glucose liquid medium, with hyphal growth unit length values decreased to 73.0 μm/tip compared to 239.8 μm/tip for the parent strain SUS2. The hyperbranching phenotype led to slightly but significantly increased cellulase secretion from 24 to 72 h in a batch culture. However, the cellulase production per unit of mycelial biomass was much more profoundly improved from 24 to 96 h.

## Introduction

Recalcitrant cellulose is a major component of plant cell wall polysaccharides, which are widely regarded as the most abundant renewable bioresource for production of bioethanol and bio-based chemicals [1]. Cellulase is a biocatalyst used to decompose cellulose into fermentable glucose and simple cello-oligosaccharides (such as cellobiose)[2]. Cellulase had long been thought to be only composed of cellobiohydrolase, endo-glucanase, and β-glucosidase [3]. In recent years, however, lytic polysaccharide mono-oxygenase (formerly GH61 endo-glucanase) was discovered to be able to boost cellulose degradation by canonical glycoside hydrolase cellulase [4]. *Trichoderma reesei* (anamorph *Hypocrea jecorina*) is a filamentous fungus serving as the main industrial microbial workhorse to produce cellulase [5]. However, its ability to produce cellulase does not parallel with the rigorous demand for low-cost cellulase in production of cellulosic ethanol and biochemicals. Therefore, there is an urgent need to improve the ability of *T. reesei* to produce higher titers of cellulase through which the cost of cellulase can be reduced.

Tremendous efforts have been carried out to improve cellulase production in *T. reesei*, through both rational genetic engineering [6] and random mutagenesis [7, 8]. Since it is well known that the regulation of cellulase expression in *T. reesei* takes place primarily at the stage of transcription [9], many trials have dealt with engineering the promoters or transcription factors [10, 11], *i.e.* the cis-and trans-elements essential for transcription, respectively. This strategy also expands to manipulating key components in signaling pathways affecting the transcription of cellulase [12, 13]. However, there are also many other aspects beyond transcription regulation that can impact cellulase secretory expression in *T. reesei*. For filamentous fungi, the secretory production of proteins is impressively related to the cell morphology due to the cell polarity [14]. Particularly, enzymes are reported to be primarily secreted at the site of actively growing hyphal tips [15] and septa [16] in filamentous fungi. Although the loci of secretion in *T. reesei* have not been reported, a highly branched hyphal morphology appears to be directly related to enhanced cellulase production in a mutant strain [17]. It can thus be assumed that the hyphal tip of *T. reesei* is likely also an important locus of protein secretion. Based on this hypothesis, engineering *T. reesei* into a hyperbranching phenotype with more hyphal tips would, therefore, be beneficial for cellulase production.

Formation of hyphal tips (*i.e.* development of cell polarity) in filamentous fungi is a complex physiological process requiring orchestrated regulation by multiple pathways and associated genes [18-21]. Although in *T. reesei* there was no literature reporting a gene regulating the mycelial development, in other fungi, many genes have already been documented for their role in formation of hyphal tips. Knocking out *COT1* encoding a serine/threonine protein kinase that belongs to the NDR family is accompanied with higher branching events in *Neurospora crassa* [22]. BarA, an acyl-CoA-dependent ceramide synthase, regulates the mycelium polarity of *Aspergillus nidulans* [23]. Deletion of the *pcl2* gene encoding a secretion pathway-specific (KEX2-like) endo-protease increases the hyphal branching frequency in *Aspergillus oryzae* [24]. Notably, these factors belong to much differing biochemical pathways and some of them, if not all, have homologous genes in *T. reesei*. This raises the possibility that by manipulating expression of the homologs of these regulatory genes, specifically RNAi-mediated gene silencing of the *Trcot1* (a homologous gene of the *N. crassa cot-1*) in this study, a phenotypic change may be observed in *T. reesei* and the consequent effects on secretory cellulase expression can be evaluated.

## Materials and Methods

### Strains, Plasmids, and Culture Conditions

The *Escherichia coli* Trans1 strain (Transgen, China) was used for plasmid construction and propagation throughout this study. The *T. reesei* SUS2 strain is an auxotroph of a mutant of QM9414 with an enhanced cellulase-producing ability [25] and is maintained in our lab. The plasmid pAPA, with the direct repeats of ampicillin resistance genes, was constructed for looping out the *pyr4* selection marker gene in the *T. reesei* transformants when needed and has been described earlier [25]. *E. coli* was cultured in Luria-Bertani (LB) medium with appropriate concentrations of ampicillin at 37°C when needed. The *T. reesei* strains were grown in the minimal medium (MM, containing (NH_4_)_2_SO_4_, 5.0 g/l; KH_2_PO_4_, 15 g/l; MgSO_4_, 0.6 g/l; CaCl_2_, 0.6 g/l; FeSO_4_·7H_2_O, 0.005 g/l; MnSO_4_·H_2_O, 0.0016 g/l; ZnSO_4_·7H_2_O, 0.0014 g/l; CoCl_2_, 0.002 g/l) supplemented with a certain kind of carbon sources (2% glucose for mycelial growth; or 2% Avicel cellulose for cellulase induction). For sporulation, *T. reesei* were grown on a potato dextrose agar (PDA) plate at 28°C.

### Plasmid Construction

The homologous gene of the *N. crassa* COT1 in *T. reesei* was identified by a BLAST search of the *T. reesei* genome. A total of ten homologous genes were found for *N. crassa* *COT1* with amino acid sequence identities ranging from 39.3% to 83.3%. The gene with the highest homology was designated *Trcot1* (Trire2:78909). The *T. reesei* SUS1 genomic DNA was extracted using a fungal DNA genome extraction kit (TianGen). The *pdc1* and *eno1* promoters and a cot1- gene fragment (49-570 bp of the first exon) were all amplified from the genomic DNA of SUS1 using the primer pairs of Pdc1-F/R, Eno1-F/R, and cot1-F/R (Table 1). To silence the *Trcot1* gene, the *pdc1* and *eno1* promoters were first ligated head-to-head in the BamHI and EcoRI restriction sites of the plasmid pAPA to obtain pAPA-pdc1p-eno1p using the Gibson assembly method [26]. This intermediate plasmid was linearized using *EcoRI* and ligated with the *Trcot1* gene fragment using the same method. This resulted in pCot1i for use in silencing the corresponding *Trcot1* gene.

### Transformation of *T. reesei*

The plasmid pCot1i was introduced into the *T. reesei* SUS2 strain by poly-ethylene glycol (PEG)-mediated chemical transformation [27]. Briefly, SUS2 was cultured in MM supplemented with 2%glucose and 10 mM uridine at 28°C for 18 h. The young mycelia were collected by filtration, and mixed with 10 mg/ml of lysing enzyme from *Trichoderma harzianum* (Sigma-Aldrich, USA). Then the mixture was incubated at 28°C with gentle shaking until large amounts of protoplasts were released. Next, 10 μg of the pCot1i plasmid was transformed into the SUS2 protoplasts and spotted on MM-glucose plates without addition of uridine at 28°C for 5-7 days. The transformants were checked by diagnostic PCR for integration of the expressing cassettes into the chromosome using the primer pairs YZ-cot1-F/R (Table 1).

### Morphological Analysis

For comparison of the growth behavior on agar plates, the spores from the parent strain SUS2 and representative transformant (2 × 10^7^/ml) were individually spotted on PDA and solid MM medium supplemented with 2% (w/v) glucose, xylan, lactose, cellobiose, xylose, or glycerol as the sole carbon source and the plates were incubated at 28°C for 72 h. The colony diameters were measured. The morphology of the *Trcot1*-silenced transformant was also compared to its parent strain in a liquid MM-glucose (2%) medium. Spores (1 × 10^7^/ml) were incubated in 250-ml Erlenmeyer flasks and the culture (50 ml) was shaken on a rotary shaker (180 rpm) for 48 h in the MM medium supplemented with 2% (w/v) glucose. The L_hgu_ (hyphal growth unit length) value was measured to quantitate the incidence of mycelial branching for the parental strain and the representative transformant. The L_hgu_ value is defined as the ratio of the total mycelium length divided by the number of tips, which is therefore an indicator of the average length of each hyphal branch (mm/tip) [24]. The hyphal length, *i.e.* distance from the branch point to septa, and the number of branches per apical or subapical compartment were measured under 40× magnification. The total length of hyphae and the number of hyphae were counted using ImageJ software (https://imagej.nih.gov/ij/). For all morphological measurements of submerged cultivations, more than 100 hyphae were measured per sample.

### Induction of Cellulase Expression in *T. reesei*

For induction of cellulase in shake flask fermentation, fresh spores (1 × 10^7^) were inoculated into 50 ml of liquid MM-glucose (2%) and shaken at 28°C for 48 h. The *cot1*-silenced transformants grew slowly and had to be cultured for 3 d for biomass accumulation. At the end of this pre-culture, the mycelia were collected and washed twice with MM with no carbon source. Then, 2 g of the mycelia were transferred into 100 ml of MM supplemented with 2% Avicel (MM-Avicel) for cellulase induction. The culture was continued at 28°C for 6 d to induce production of cellulases. From 24 to 144 h post induction, 2 ml of the culture supernatants was periodically collected for assay of the cellulase activity, extracellular protein concentration, and mycelial biomass. The inducing culture contains insoluble cellulose Avicel, which prohibited us from directly measuring the mycelia weight. Therefore, we determined the mycelia protein according to the method described by Jayaraman [28] as a representative of the fungal biomass.

### Assay of Enzymatic Activities and Protein Concentration

For the overall cellulase activity, the reaction used one strip of Whatman No.1 filter paper (6 × 1 cm) as the substrate and 100 μl of the culture supernatant as the crude enzyme in 50 mM acetate buffer (pH 4.8). The mixture (1 ml in total) was incubated at 50°C for 1 h. The released reducing sugars were determined using the 3,5-dinitro-salicylic acid (DNS) method [29] and the OD_540_ of the reactions was measured. One unit of the overall cellulase activity was defined as the amount of enzyme that released 1 μmol of reducing sugar per hour under the assay conditions. To determine the endo-glucanase activity, 900 μl of 1% (w/v) sodium carboxymethyl cellulose (CMC-Na) was instead used as the substrate. The reaction was incubated at 50°C for 10 min. One unit of endo-glucanase activity was defined as the amount of enzyme that released 1 μmol of reducing sugar per minute under the assay conditions. The β-glucosidase activity was determined using *p*-nitrophenol-β-D-glucopyranoside (*p*NPG) as the substrate. The reaction consisted of 100 μl of appropriately diluted enzymes and 400 μl of 1.25 mM *p*NPG dissolved in McIlvaine buffer (200 mM Na_2_HPO_4_, 100 mM citric acid, pH 4.8). The mixture was incubated at 50 °C for 10 min. One unit of β-glucosidase activity was defined as the amount of enzyme that released 1 μmol of *p*-nitrophenol in one minute. The protein concentration of the fermentation supernatants was determined using a BCA-200 Protein Assay Kit (Pierce, USA) following the instruction of the manufacturer.

### Quantitative Reverse Transcription PCR Analysis

For quantitative reverse transcription PCR (qRT-PCR), the mycelia of SUS2 strain and its *Trcot1*-silenced transformant cultured in MM-glucose (2%) for 24 h were collected. The mycelia were quickly frozen in liquid nitrogen and pulverized using a pestle and mortar. Using the TRIzol reagent (Thermo Fisher Scientific, USA), the total RNA was isolated from the pulverized mycelia. One microgram of the total RNA was reverse-transcribed to cDNA using a First Strand cDNA Maxima Synthesis Kit (TOYOBO, China). Then, using the *actin* gene as an endogenous reference gene, qRT-PCR was performed in an Applied Biosystems QuantStudio 6 Flex Real-Time PCR System (Applied Biosystems, USA) using a TransScript Green One-Step SuperMix (TransGen). The primers used in qRT-PCR were listed in Table 1. The qRT-PCR was performed with the following steps: initial denaturation at 95°C for 10 min, then 40 cycles of 94°C for 30 sec, 60°C for 20 sec, and 72°C for 20 sec.

## Results

### Construction of the Plasmid for RNAi-Mediated Silencing of *Trcot1*

**Fig. 1:**
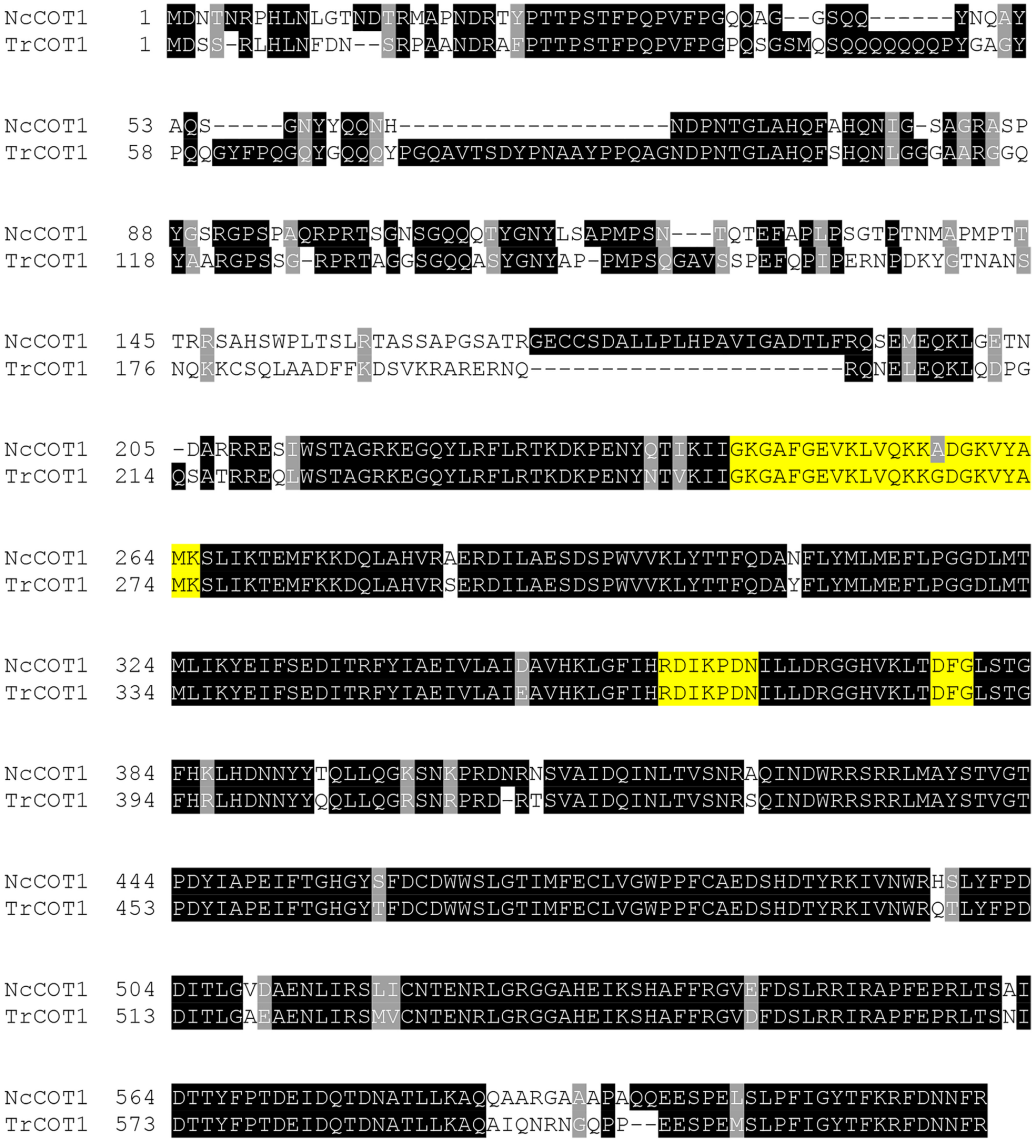
The amino acids shaded in black are conserved residues, while those in grey indicate similar ones. The amino acis in yellow indicate the glycine-rich loop containing the MgATP binding site (Gly253 -Lys275), the catalytic loop (Arg368-Asn374), and the carboxyl groups participating in substrate recognition (Asp387-Gly389).

Through a BLAST search, a close homolog of *NcCOT1* was identified in the *T. reesei* genome. An amino acid sequence alignment of *Tr*COT1 with *Nc*COT1 was given in Fig. 1. *Tr*COT1 is highly similar to *Nc*COT1, sharing an amino acid sequence identity of 83.3%. The functional sites critical for catalysis are conserved in *Tr*COT1. These include the glycine-rich loop containing the MgATP binding site (Gly253 -Lys275), the catalytic loop (Arg368-Asn374), and the carboxyl groups participating in substrate recognition (Asp387-Gly389) [30, 31]. These suggested that *Tr*COT1 is also a serine/threonine kinase and could play a regulatory role in *T. reesei*.

**Fig. 2:**
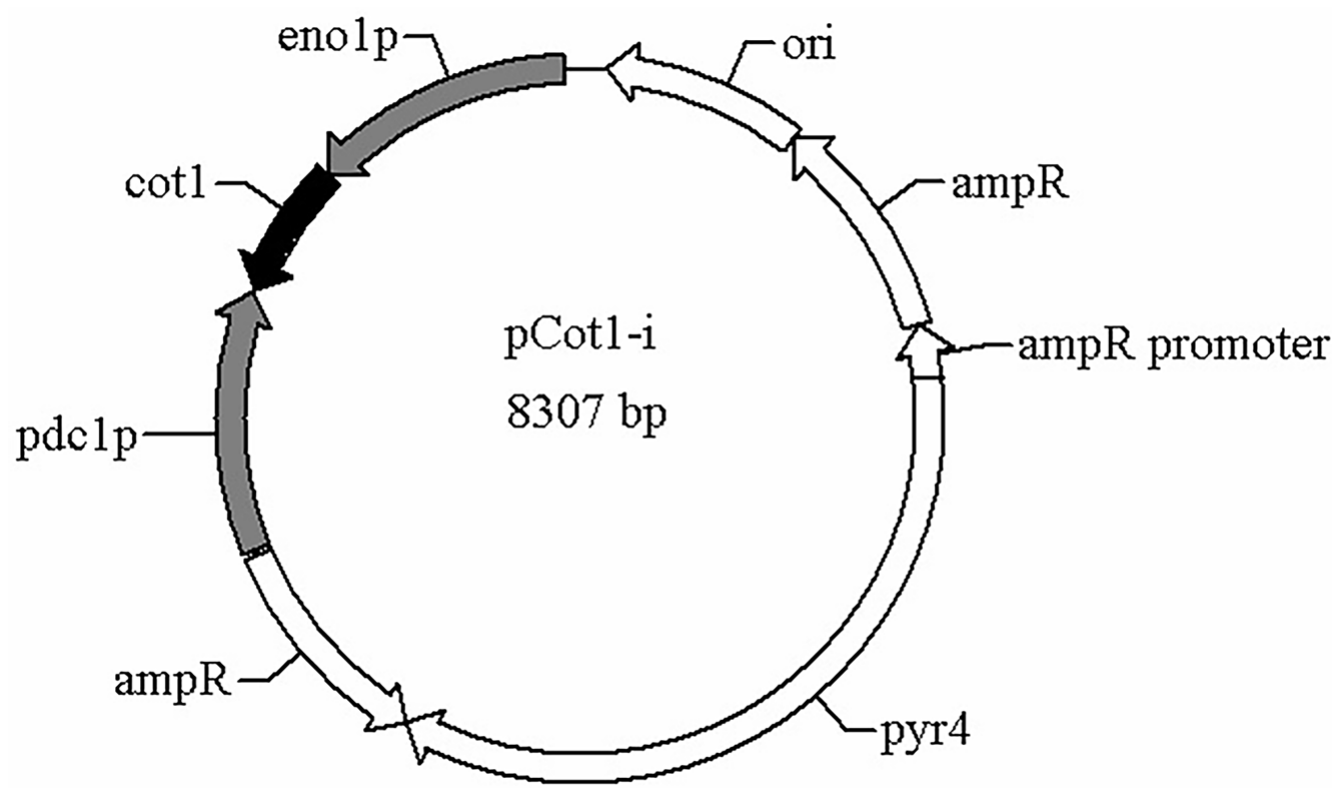
Schematic diagram of the plasmid pCot1i used for RNAi-mediated gene silencing of *Trcot1* in *T. reesei*. The key elements are as following: pdc1p, the *pdc1* promoter; eno1p, the *eno1* promoter; cot1, the *cot1* gene fragment; pyr4, the expressing cassette for the *pyr4* gene; ampr, the ampicillin resistance gene; ori, the plasmid replication origin motif.

For analysis of the role of *Trcot1* in regulating the morphological development of mycelia, a strategy via RNAi-mediated repression of gene expression was employed. The strong and constitutive *pdc1* and *eno1* promoters amplified from the *T. reesei* genomic DNA were assembled to obtain an intermediate plasmid, in which a gene fragment from the first exon of the *Trcot1* gene (49-570 bp) was inserted between the two promoters (Fig. 2). The head-to-head dual promoters allow the transcription of the *Trcot1* gene from two opposite directions, leading to formation of a double-stranded RNA in vivo. This double-stranded RNA is supposed to be recognized by RNA-induced silencing complex (RISC) and cleaved by Dicer proteins into small RNA fragments with lengths of 21-25 nt, which direct the cleavage of the target mRNA(s) [32]. The integrity of the recombinant plasmid was verified by both restriction digestion and DNA sequencing (data not shown).

### The RNAi-Silenced Transformant Was Largely Different in Colony Morphology

The plasmid for RNAi-mediated gene silencing of *Trcot1* (pCot1-i) was transformed into *T. reesei* and the integration of the RNAi cassette in the chromosome of transformants was verified by PCR (data not shown). From 48 h of these PCR-selected transformants, after 20 h they exhibited an appreciable change of the colony morphology on PDA plates compared with their parent strain, suggesting that the silencing of *Trcot1* can affect the growth of these strains. One representative transformant, designated SUS2/Trcot1i, was selected for further analyses. Total RNA was extracted from the mycelia collected after 24 h of culture in the MM-glucose medium and the transcript levels of *Trcot1* of the parent strain and the transformant were quantified by RT-qPCR. The transcript abundance of *Trcot1* in SUS2/TrCot1i was 39.8 % lower than that of SUS2 (Table 2). This clearly indicated that the expression of the *Trcot1* was significantly repressed by RNAi-mediated gene silencing.

**Fig. 3:**
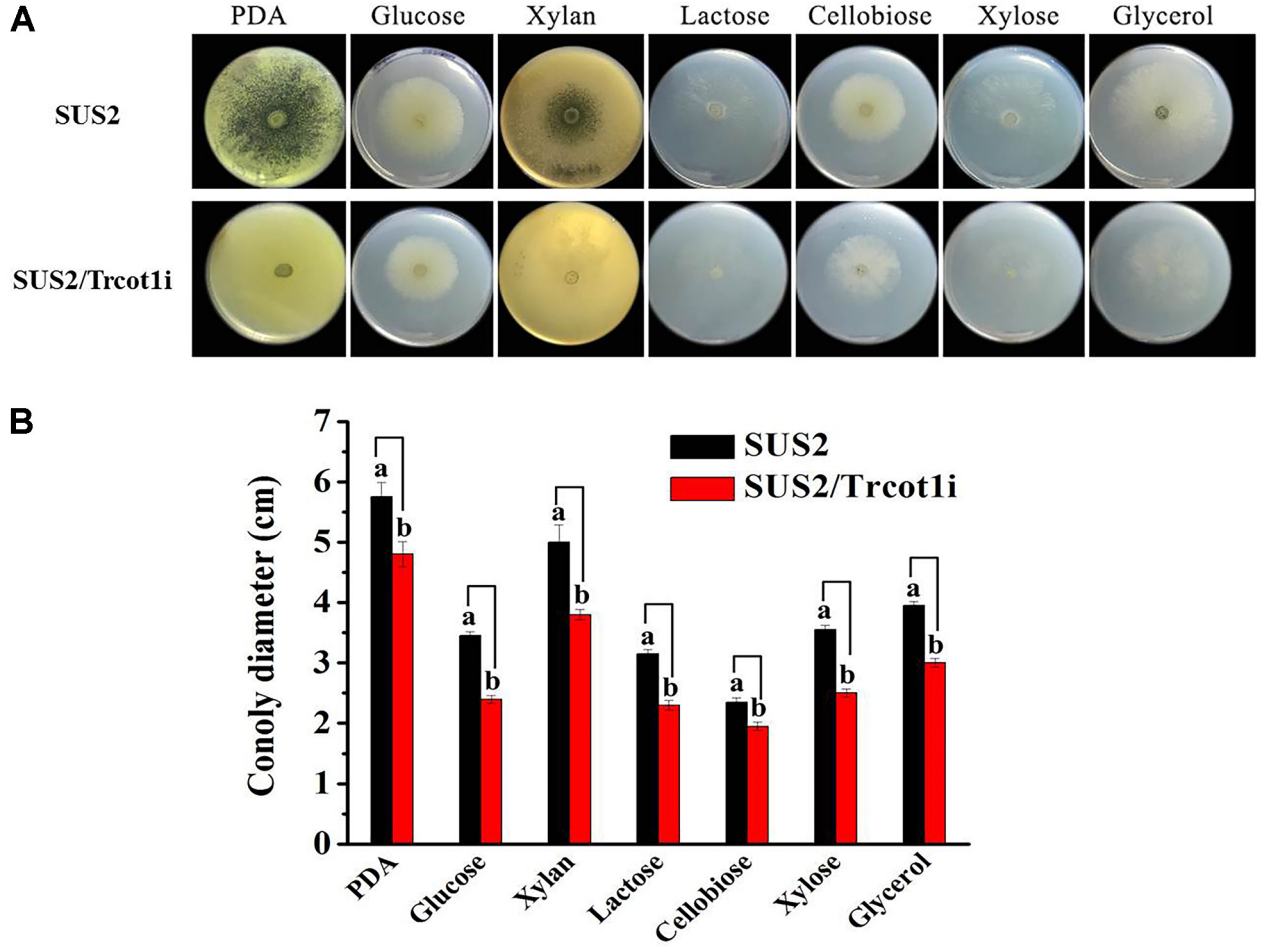
Effects of *Trcot1* silencing on SUS2/Trcot1i growth on solid agar plates. (**A**) The colony morphology; (**B**) quantitation of the colony diameters. PDA: potato dextrose agar. Glucose, xylan, lactose, cellobiose, xylose, and glycerol are minimal media containing one of these carbohydrates as the sole carbon source. Different letters (a and b) for each same-culture medium mean that there are significant differences between the colony diameters (*p* < 0.05).

As shown in Fig. 3A, the sporulation of the transformant SUS2/Trcot1i on the PDA plate was much slower than that of the host strain. The colony diameters of SUS2/Trcot1i on the MM-agar plates containing glucose, xylan, lactose, xylose, or glycerol as the sole carbon source were smaller than that of the wild type. Although the colony diameter did not change on the MM-cellobiose medium, the mycelial density was apparently lower than that of the wild type. The quantitation of the colony diameters of the two strains on different culture medium was given in Fig. 3B, which indicated 17.0~30.4% reduction in the colony size in MM with different carbon source. These results demonstrated that the growth of the transformant was significantly retarded.

### The RNAi-Silenced Transformant Displayed a Hyperbranching Phenotype 

**Fig. 4:**
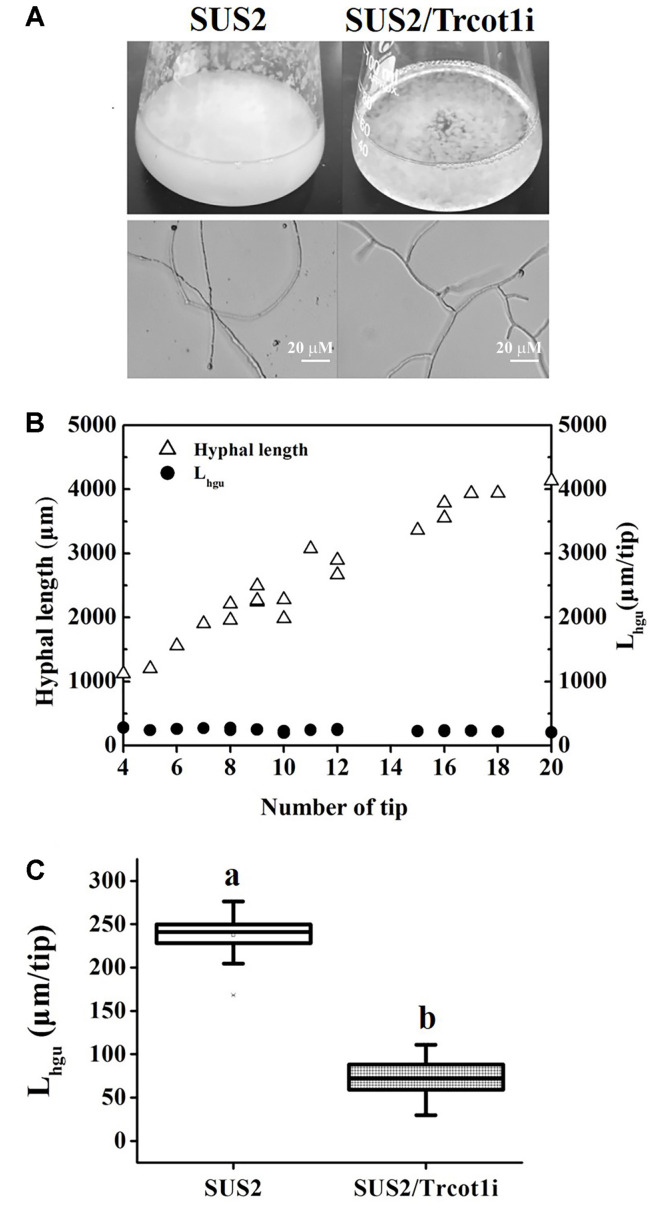
Effects of *Trcot1* silencing on hyphal branching. (**A**) *T. reesei* grown in shake flasks containing the MM-glucose liquid medium as observed by naked eyes and under a microscope. Each scale bar represents 20 μm. (**B**) Demonstration of L_hgu_ as a constant value using SUS2 as a model strain. C: a comparison of L_hgu_ values of the SUS2 and SUS2/Trcot1i. Different letters (a and b) mean that there are significant differences between the L_hgu_ values (*p* < 0.05).

In submerged shake flask fermentation, SUS2 accumulated large amounts of mycelia after 48 h of culture. However, many fewer mycelia were observed for SUS2/Trcot1i (Fig.4A). The mycelia of SUS2/Trcot1i formed compact globules suspended in the culture broth. Observing the mycelia under microscope suggested that SUS2/Trcot1i had a hyperbranching phenotype (Fig. 4A). In order to ascertain whether the interference of *Trcot1* expression would indeed result in a hyperbranching phenotype, the L_hgu_ value was measured in exponentially growing batch cultivations of SUS2/Trcot1i and its parent strain. L_hgu_ is an indicator of the degree of mycelium branching, with a lower L_hgu_ value indicating a more branched phenotype [24]. Using the SUS2 as an example, it was demonstrated that, although the total lengths of different mycelial units in the same strain were not the same, the corresponding L_hgu_ values remained as a constant (Fig. 4B), indicating that the L_hgu_ value could be used to measure and compare the branching morphological characteristics of different *T. reesei* strains. It was thus discovered that the RNAi-cassettes-bearing transformant SUS2/Trcot1i had a much smaller L_hgu_ value of 73.0 μm/tip than that of the SUS2 (239.8 μm/tip, Fig. 4C). The results undoubtedly demonstrated that the *Trcot1*-silenced strain was comparably more highly branched.

**Fig. 5:**
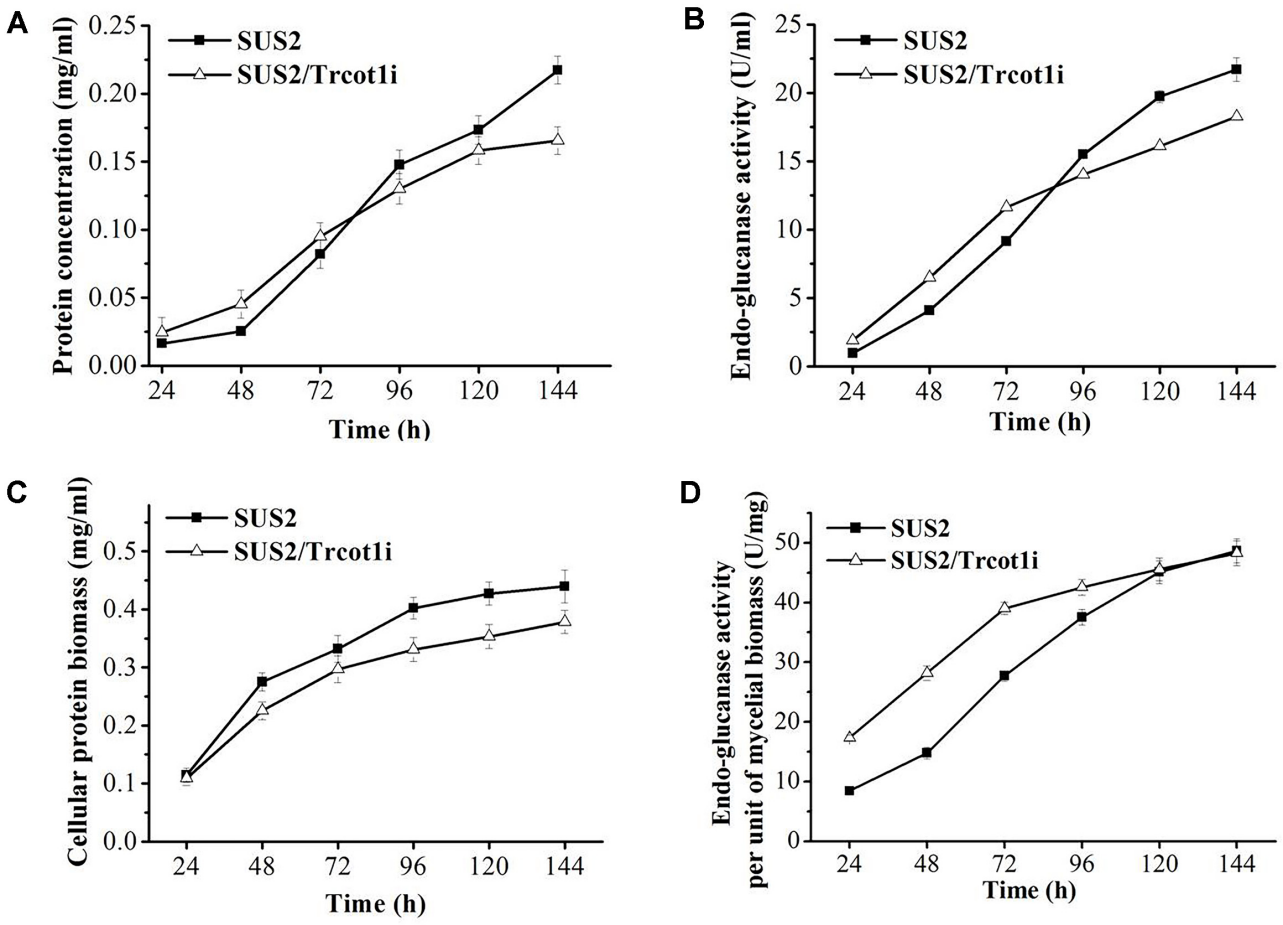
Cellulase expression in SUS2 and SUS2/Trcot1i. (**A**) Extracellular protein concentration. (**B**) Endo-glucanase activity. (**C**) The mycelial biomass as reflected by NaOH-extracted cellular protein concentration. (**D**) Endo-glucanase activity per mg of mycelial protein biomass.

Cellulase Production in the RNAi-Silenced Transformant Since the hyperbranching phenotype of filamentous fungi has been reported to be positively related with higher amounts of secreted enzymes [8, 33], it was therefore asked if the secreted cellulase and extracellular protein concentration would increase in the RNAi-silenced hyperbranching transformant SUS2/Trcot1i. In the early stage of the culture (from 24 to 72 h), the extracellular protein concentration and the endo-glucanase activity of SUS2/Trcot1i were slightly higher than those of the parent strain SUS2. After 72 h, the protein concentration and endo-glucanase activity of SUS2/Trcot1i were outperformed by the SUS2 parent strain (Figs. 5A and 5B). SUS2/Trcot1i grew slower than its parent strain in solid and liquid media (Figs. 3 and 4A). Therefore, it was asked if the cellulase production per unit of mycelial biomass had changed. SUS2/Trcot1i clearly showed a considerable decrease in mycelial biomass accumulation (Fig. 5C). From 24 h to 144 h of cultivation, the mycelia biomass of SUS2/Trcot1i (as reflected by concentration of NaOH-extracted soluble cellular protein) amounted to 0.11~0.38 mg/ml, while the values were 0.11~0.44 mg/ml for the parent strain SUS2. As a result, the endo-glucanase activity per unit of mycelial biomass of SUS2/Trcot1i was 17.3~42.6 U/mg from 24 h to 96 h post cellulose induction, significantly higher than those of SUS2 (8.4~37.5 U/mg) (Fig. 5D). It was determined by RT-qPCR that the transcript abundance of the major cellulase genes cbh1, cbh2, egl2 and bgl1 was not affected in SUS2/Trcot1i (Table 2).

## Discussion

Secretory expression of cellulase in *T. reesei* is a very complex process involving coordinated regulation of transcription, translation, secretion, proteolysis, and post-translation modifications. Adding to this complexity is the multicellular nature of *T. reesei* as a filamentous fungus. Unlike the unicellular microbes such as *Saccharomyces cerevisiae* and *Pichia pastoris*, the cellular morphology of *T. reesei* has a large impact on secretory protein expression. The establishment of cell polarity, by itself, is also a complicated process entailing genes distributed in diverse pathways [34]. This wealth of associated genes provides a good opportunity to find a gene which, upon functional disruption, may lead to a hyperbranching phenotype.

In *N. crassa*, a genome-wide gene knockout mutant library is commercially available, which allows rapid screening of a mutant with desirable phenotype due to specified regulatory function [35]. This resulted in identification of *gul-1* as a regulatory gene of cell polarity in *N. crassa* [36]. In contrast, there is no such mutant library in *T. reesei*. Therefore, we instead chose one regulatory gene from *N. crassa* with the defined function in determining cell polarity and investigated if disrupting the expression of its homologous gene in *T. reesei* would affect the morphology of the cell. The RNAi-mediated gene silencing strategy was used instead of gene knockout because the strain used in this study is resistant to homologous gene replacement and CRISPR/Cas9-mediated gene disruption (data not shown). One additional advantage is that RNAi can be conveniently used for a gene when its knockout leads to cell death.

The *N. crassa* COT1 belongs to the NDR kinase, which is important in regulating cell cycle and morphogenesis [37]. NcCOT1 genetically interacts with the mitogen-activated protein kinases MAK1 and MAK2 [22] and may modulate actin dynamics [38]. Both protein kinases have close homologs in *T. reesei* (Trire2:82351 for MAK1 and Trire2:121539 for MAK2, sharing amino acid sequence identities of 90.3% and 94.5%, respectively). In general, disruption of *Trcot1* reduced the size of the mutant colony on solid plates on a rich medium (PDA) and the minimal media containing one of the sugar or glycerol carbon sources (Fig. 3). The appreciable change of mutant colonies strongly suggested that the cell morphology has been modified and this was ascertained by microscopic observation of the fungal mycelia cultured in liquid medium. The SUS2/Trcot1i mutant was of a hyperbranching phenotype and its biomass accumulation was lower than that of the wild type. Since the residues important for catalysis are well conserved in *Tr*COT1 (Fig. 1), it could be concluded that *Tr*COT1 indeed plays a role in regulating the cell polarity in *T. reesei*. Interestingly, there are nine more homologs of NcCOT1 in *T. reesei* with amino acid sequence identities of 39.3%-46.9%. All of them are annotated as putative protein kinases. However, whether they play a regulatory role in *T. reesei* and, specifically in cell morphogenesis, remains to be unveiled.

In *T. reesei*, the relationship of a gene function to the hyperbranching phenotype is seldom investigated. Our study thus provides an example of how to obtain a hyperbranching phenotype through genetic engineering in *T. reesei*. Although functional disruption of cot-1 leads to hyperbranching in *N. crassa*, it is not known if this phenotype is associated with more cellulase production [37]. In *A. oryzae*, the *pcl2*-deleted mutant has a hyperbranching phenotype, corresponding to an enhanced capability to secrete more enzymes [24]. However, disruption of a chitin synthase B increased the hypha branching but not a-amylase production in *A. oryzae* [39]. In our study, the hyperbranching phenotype slightly but significantly increased endo-glucanase secretion from 24 to 72 h. More importantly, the endo-glucanase activity per unit of mycelial biomass was much improved in the *Trcot1*-silenced strain from 24 to 96 h. The retarded fungal growth negatively impacted on apparent cellulase production in SUS2/Trcot1i.

The discrepancy in effects of hyperbranching on secretory protein production from our study and those of other researchers suggested that the choice of different regulatory genes may have profound effects on the ultimate protein secretion, even when the desired hyperbranching phenotype is successfully obtained. This further indicated that the genetic background of a strain could interact with the mutation. The beneficial effects incurred by increased hyphal tip numbers may be compromised by significantly decreased number of mycelia cells. To overcome this undesirable effect, on one hand, the genes involved in regulating cell polarity distributed in diverse pathways can be carefully screened until a gene whose manipulation (functional disruption or overexpression) leads to hyper-branching while not negatively affecting mycelial biomass accumulation. On the other hand, genetic engineering of other cellular genes to improve biomass accumulation, while maintaining the hyperbranching phenotype induced by *Trcot1* silencing, is another route towards successfully obtaining cellulase hyperproducers.

## Figures and Tables

**Table 1:** Primers used in this study.

Primer	Sequences (5′→3′)^a^
cot1-F	CGCAGCTACAGCACAATCGAATTCGTCCTTGAAGAAGTCT
cot1-R	CTGAAATAGCTTCAAAGCCAACGATCGAGCCT
Pdc1-F	ATCACTAGTTCTAGAGCGGCCGCCGATGAAAGCCTTGCA
Pdc1-R	CTGAAATAGCTTCAAAGAATTCGATTGTGCTGTAGCT
Eno1-F	AGCTACAGCACAATCGAATTCTTTGAAGCTATTTCA
Eno1-R	TCATTACCAATTGGCGCGCCTTCTCAAATACCGCA
YZ-cot1-F	CTTCTTCATCAACCACC
YZ-cot1-R	GCCAGGCCGTCACCAGC
qActin-F	TGAGAGCGGTGGTATCCACG
qActin- R	GGTACCACCAGACATGACAATGTTG
qcot1-F	CTTGCCGGGTGGAGATTTGA
qcot1- R	TCGCTGTTTGCAAGGTCAGT
RTQcbh1F	GCTGCCGGTGCGGCTTGAAC
RTQcbh1R	CTGGCCATTGATGAACTTCAGATCGC
RTQcbh2F	CGTCAAATTGTCGTGGAA
RTQcbh2R	ACTGAGCATTGGCACACTT
RTQegl2F	CTACCTCAACAAGCTCATCAA
RTQegl2R	TCTTCAACGGAGGATAAACC
RTQbgl1F	AGTGACAGCTTCAGCGAG
RTQbgl1R	GGAGAGGCGTGAGTAGTTG

^a^The underlined regions are for homologous recombination.

**Table 2:** Relative transcript abundance of selected genes in SUS2 and SUS2/Trcot1i.

Gene	Relative transcript abundance	

	SUS2	SUS2/Trcot1i
*cot1*	1.0	0.6±0.1
cbh1	1.0	1.1±0.1
cbh2	1.0	1.1±0.2
egl2	1.0	1.1±0.1
bgl1	1.0	1.1±0.1
